# Unicompartmental knee replacement and high tibial osteotomy for medial unicompartmental knee osteoarthritis: A comparative study protocol

**DOI:** 10.1097/MD.0000000000023454

**Published:** 2020-12-04

**Authors:** Yongqiang Yin, Xu Zhang, Kaiming Zhang, Xiang He

**Affiliations:** Department of Orthopedics, Yueyang Second People's Hospital, Hunan Province, China.

**Keywords:** high tibial osteotomy, medial unicompartmental knee osteoarthritis, retrospective, study protocol, unicompartmental knee replacement

## Abstract

**Background:**

Many clinical studies have been published in the literature to compare the outcomes of unicompartmental knee replacement (UKR) and high tibial osteotomy (HTO), but reached different conclusions. Therefore, the relative merits and demerits of these 2 procedures remain under debate. The purpose of the present protocol was to design a retrospective comparative study to further investigate the clinical effectiveness of HTO compared to UKR in the medial unicompartmental osteoarthritis of knee patients.

**Methods:**

This is an observational retrospective research, which prospectively collected the data from several surgeons in our center and utilized the above 2 methods to treat the unicompartmental osteoarthritis of knee. In our single hospital, we reviewed unicompartmental knee osteoarthritis patients treated using UKR or HTO from June 2016 to February 2018. For the HTO, its criteria included:

1. patients ≤65 years of age with separated medial compartment osteoarthritis of knee joint, and the patients’ age is equal to or less than 65 years old,
2. patients without the ligament instability.

For the UKR, its inclusion criteria contained

1. the osteoarthritis of knee joint, including the isolated medial compartments of knee, but no degenerative changes in lateral compartment,
2. rectifiable varus deformity, and
3. the patients with an intact anterior cruciate ligament.

In our cohorts, the clinical investigations of the knee were composed of the objective parameters, which were recorded and then documented through utilizing the Function Score and Orthopedic American Knee Society Knee Score. The extra clinical findings evaluated involved operative time, postoperative requirements of blood transfusion, possible postoperative complications, as well as the range of motion.

**Conclusion and discussion:**

The results of this study will provide clinical evidence on appropriate surgical treatment for patients with medial unicompartmental knee osteoarthritis.

**Trial registration:**

This study protocol was registered in Research Registry (researchregistry6152).

## 1 Introduction

Knee osteoarthritis is a kind of familiar disease, which is characterized via the limitation of the recreational and physical activities in the elderly. More than half of people over 65 have osteoarthritis.^[1]^ Although knee osteoarthritis may affect any or all 3 knee compartments, one third of the patients are influenced in just 1 compartment. In up to 50% of patients, the changes of knee arthritis mainly occur in medial compartment, with fewer changes occurring on the lateral side or patella-femoral compartment.^[2–6]^

At present, the 2 most familiar surgical methods to treat the medial knee osteoarthritis are the medial unicompartmental knee replacement (UKR) and the medial opening wedge high tibial osteotomy (HTO). For the UKR, the traditional indications involve the unilateral osteoarthritis of knee, older than 60 years old, deformity angle less than 15°, low functional requirements, and weight less than 82 kg.^[7–11]^ As a method of treating the unilateral osteoarthritis of knee, UKR has significant advantages in comparison with the total knee arthroplasty, for instance, less loss of blood during operation, retaining the unaffected side, faster time of recovery, refined functional prognosis, and lower incidence rate during perioperative period.^[12–17]^ HTO is a globally recognized choice for treating the medial osteoarthritis of the knee, particularly in active and young patients. Patients receiving medial opening wedge high tibial osteotomy benefit from protection of natural joint and the physical load is nearly completely unaffected.^[18,19]^

Many clinical studies have been published in the literature to compare the outcomes of UKR and HTO, but reached different conclusions.^[20,21]^ Therefore, the relative merits and demerits of these 2 procedures remain under debate. The purpose of the present protocol was to design a retrospective comparative study to further investigate the clinical effectiveness of HTO compared to UKR in the medial unicompartmental osteoarthritis of knee patients.

## 2 Materials and methods

### 2.1 Study design and population

This is an observational retrospective research, which prospectively collected the data from several surgeons in our center and utilized the above 2 methods to treat the unicompartmental osteoarthritis of knee. In our single hospital, we reviewed unicompartmental knee osteoarthritis patients treated using UKR or HTO from June 2016 to February 2018. Our experiment was approved via the Institutional Review Board of the Yueyang Second People's Hospital. The present protocol is also registered in Research Registry (researchregistry6152).

For the HTO, its criteria included:

1. patients ≤65 years of age with separated medial compartment osteoarthritis of knee joint, and the patients age is equal to or less than 65 years old,
2. patients without the ligament instability.

For the UKR, its inclusion criteria contained

1. the osteoarthritis of knee joint, including the isolated medial compartments of knee, but no degenerative changes in lateral compartment,
2. rectifiable varus deformity, and
3. the patients with an intact anterior cruciate ligament.

Patients with the following situations would be excluded: patients diagnosed with the traumatic osteoarthritis of knee, osteonecrosis or inflammatory arthritis (for instance, rheumatoid arthritis), symptomatic osteoarthritis of knee joint in the patellofemoral joint or lateral ventricle, patients with the history of knee infection, the patients who refused to take part in or the patients were unable to evaluate the clinical results during the follow-up period of 24 months.

### 2.2 Surgical procedures and postoperative rehabilitation

All the operations were carried out through several experienced surgeons in a standard manner under the condition of general anesthesia. The UKR was implemented using fixed-bearing, metal-backed, and cemented components in a standard manner (Zimmer Biomet, Warsaw, IN, USA). The HTO was conducted through the medial opening-wedge osteotomy fixed with screws and a plate (DePuySynthes, Oberdorf, Switzerland). At the reporting dates, there were no significant changes in surgical techniques of UKR and HTO.

For the postoperative rehabilitation, UKR patients were allowed weight-bearing within the tolerance range, and to use crutches or walker when needed, and allowed the range of motion. Patients with HTO received the protective weight-bearing treatment for 4 weeks to 6 weeks. The range of motion was allowed under tolerable conditions, and movement was allowed in the locked knee bracket until quadriceps function returned to normal. At 6 weeks, the progressive loading was allowed and the radiographs were satisfactory. In the above 2 groups, the progressive activities could be conducted after 3 months as tolerated.

### 2.3 Outcome evaluation

The records of patient were reviewed to collect the following information: the anesthesia type, smoking status, and the scores of Chalson Comorbidity Index and American Society of Anesthesiologists, the level of hospital activity, as well as the perioperative thromboprophylaxis. In our cohorts, the clinical investigations of the knee were composed of the objective parameters, which were recorded and then documented through utilizing the Function Score and Orthopedic American Knee Society Knee Score. These assessments were performed preoperatively and postoperatively, and were repeated during postoperative follow-up. The extra clinical findings evaluated involved operative time, postoperative requirements of blood transfusion, possible postoperative complications, as well as the range of motion (Table 1).

**Table 1:** Outcomes.

Outcomes	Group A	Group B	*P* value
KSKS			
KSFS			
Operative time			
Blood transfusion			
Complications			
Range of motion			

KSFS = Knee Society Function Score, KSKS = Knee Society Knee Score.

### 2.4 Statistical analysis

Results were expressed as the standard deviations and means. Through utilizing the Mann–Whitney *U* test, the analysis of continuous variables could be carried out, and with the Chi-Squared test (or using the Fisher exact test, if appropriate), the categorical data could be analyzed for both independent samples. All the statistical analyses could be carried out via the software of lSPSS ver. 21.0 program (SPSS, Inc., Chicago, IL). *P* value less than .05 indicates that there is statistical significance.

## 3 Discussion

The objective of unicompartmental osteoarthritis of knee surgery is to reduce pain, restore related functions and then improve the patients life quality. A recent study comparing the clinical outcomes of UKR and HTO with a follow-up of 4 years showed that UKR can quickly restore the function of the knee joint, thus it is a reasonable alternative to HTO in treating the medial osteoarthritis of knee joint. Unfortunately, despite improvements in surgical techniques and modern repair designs, not all patients receiving UKR can achieve excellent clinical results. The combination of multiple factors, such as inappropriate patient selection and misalignment techniques between surgeons performing lower UKR volumes, may explain the poor clinical results of UKR. The purpose of the present protocol was to design a retrospective comparative study to further investigate the clinical effectiveness of HTO compared to UKR in patients with medial unicompartmental knee osteoarthritis.

## Author contributions

Yongqiang Yin conceived, designed, and planed the study. Yongqiang Yin, Xu Zhang, and Kaiming Zhang are recruiting the study participants and performing the interventions. Xiang He supervised the study. Yongqiang Yin and Kaiming Zhang will interpret and analyze the data. Yongqiang Yin drafted the manuscript. Xu Zhang critically revised the manuscript for important intellectual content. All authors have full access to the manuscript and take responsibility for the study design. All authors have approved the manuscript and agree with submission.

**Conceptualization:** Yongqiang Yin, Xu Zhang.

**Data curation:** Yongqiang Yin, Xu Zhang.

**Formal analysis:** Yongqiang Yin.

**Investigation:** Xu Zhang, Kaiming Zhang.

**Methodology:** Xu Zhang, Kaiming Zhang, Xiang He.

**Project administration:** Yongqiang Yin.

**Software:** Xu Zhang.

**Supervision:** Xiang He.

**Visualization:** Kaiming Zhang.

**Writing – original draft:** Yongqiang Yin, Xu Zhang.

**Writing – review & editing:** Kaiming Zhang, Xiang He.
